# Supplementary material for: Evaluating soluble Axl as a biomarker for glioblastoma: A pilot study
Source: PLoS One. 2024 Jul 5;19(7):e0301739. doi: 10.1371/journal.pone.0301739 (PMC11226040; doi:10.1371/journal.pone.0301739)
Supplement: S1 Raw images — (PDF) [file pone.0301739.s001.pdf]

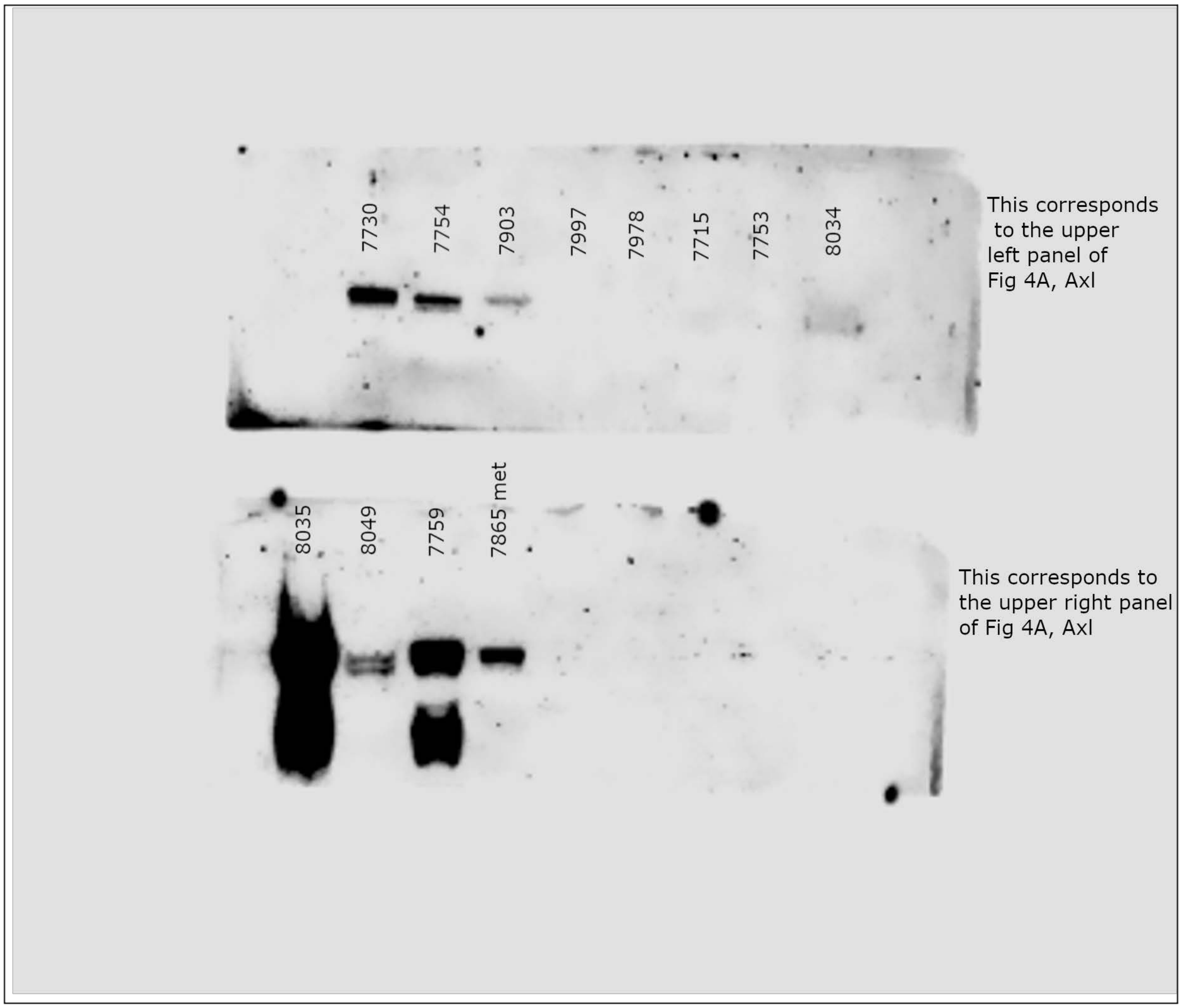
7730
7754
7903
7997
7978
7715
7753
8034
This corresponds
to the upper
left panel of
Fig 4A, Axl
8035
8049
7759
7865 met
This corresponds to
the upper right panel
of Fig 4A, Axl

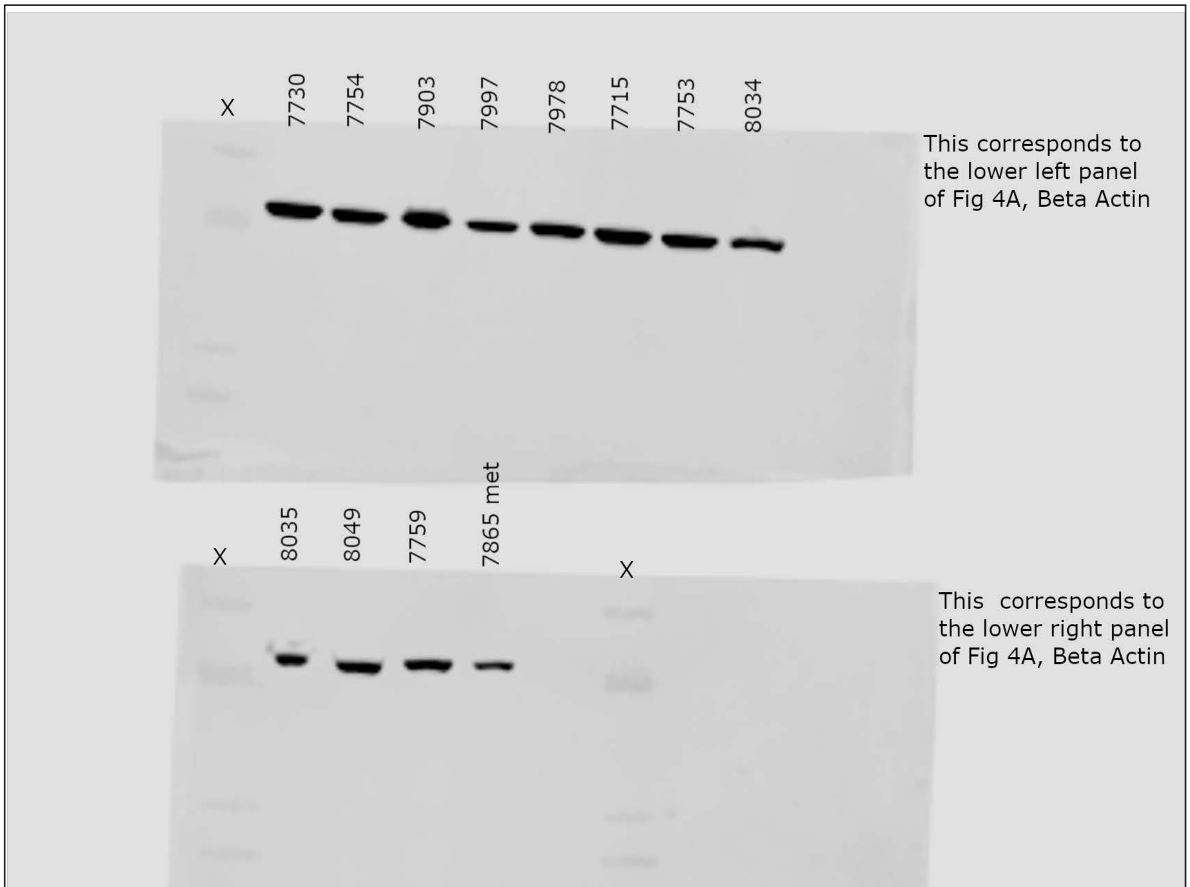
X
7730
7754
7903
7997
7978
7715
7753
8034
This corresponds to
the lower left panel
of Fig 4A, Beta Actin
X
8035
8049
7759
7865 met
X
This corresponds to
the lower right panel
of Fig 4A, Beta Actin
